# Clinical impact of the TPM1 p.Tyr221Cys variant causing hypertrophic cardiomyopathy

**DOI:** 10.1016/j.gendis.2025.101575

**Published:** 2025-02-26

**Authors:** Alessio Marinelli, Giuliana Longo, Vincenzo Cirigliano, Esther Campopiano, Clementina Dugo, Luca Ghiselli, Andrea Chiampan, Gianluca Ferri, Alessandro Costa, Stefano Bonapace, Laura Lanzoni, Giulio Molon

**Affiliations:** a IRCCS Sacro Cuore Don Calabria, Don Angelo Sempreboni street n.5, Negrar di Valpolicella 37024, Italy; b Veritas Intercontinental, C/Orense 58, 2º, Madrid 28020, Spain; c Villa Sofia-Cervello United Hospitals, Villa Sofia Cardiology Department, Piazza Salerno 1, Palermo 90146, Italy

Hypertrophic cardiomyopathy (HCM) is a relatively common (about 1 in 500 in the general population) inherited cardiac condition characterized by hypertrophy of the left ventricle wall, usually the septum, not explained by other conditions. The most severe forms can be responsible for sudden cardiac death.

Offspring carriers have a 50% chance of inheriting the same disease-causing genetic variant.^1^^,^^2^ Genotype-positive family members can be targeted for ongoing cardiac screening while genotype-negative relatives can be released from life-long surveillance. Next-generation sequencing led to an expansion in the number of genes included in diagnostic gene panels. However, the identification of variants in genes with limited gene–disease association, variants of uncertain significance, has the potential to add uncertainty and misinterpretation.

Here, we report the possible “likely-pathogenetic” role of a TPM1 variant, up to now classified as a variant of uncertain significance, in a patient with HCM.

A 66-year-old female was referred to our inherited cardiac condition clinic. Despite a significant family history, she had a diagnosis of HCM during hospital admission for other causes (low urinary tract infections). Due to abnormal findings on the electrocardiogram (Fig. 1B), an echocardiogram was performed showing a thicker/asymmetric septum. The HCM features were confirmed by cardiac magnetic resonance, which showed a septum of 25 mm with patchy late enhancement and dilated left atrium (Fig. 1A). The family history was clinically significant, both for family history of sudden cardiac death (mother and grand-mother deceased suddenly at 49 years old and 44 years old, respectively), and other four family members affected by HCM (patient's daughter, sister, and her two sons). The other five brothers, all of them older than the two sisters affected, had no diagnosis of HCM (Fig. 1C). Clinical presentation age in the proband affected relatives was around their 30s. Accordingly to the numerical predictor HCM-risk-score introduced by the European Society of Cardiology, the patient was implanted with a dual chamber implantable cardioverter defibrillator. The other two family members were already implanted with such a defibrillator.

**Figure 1:**
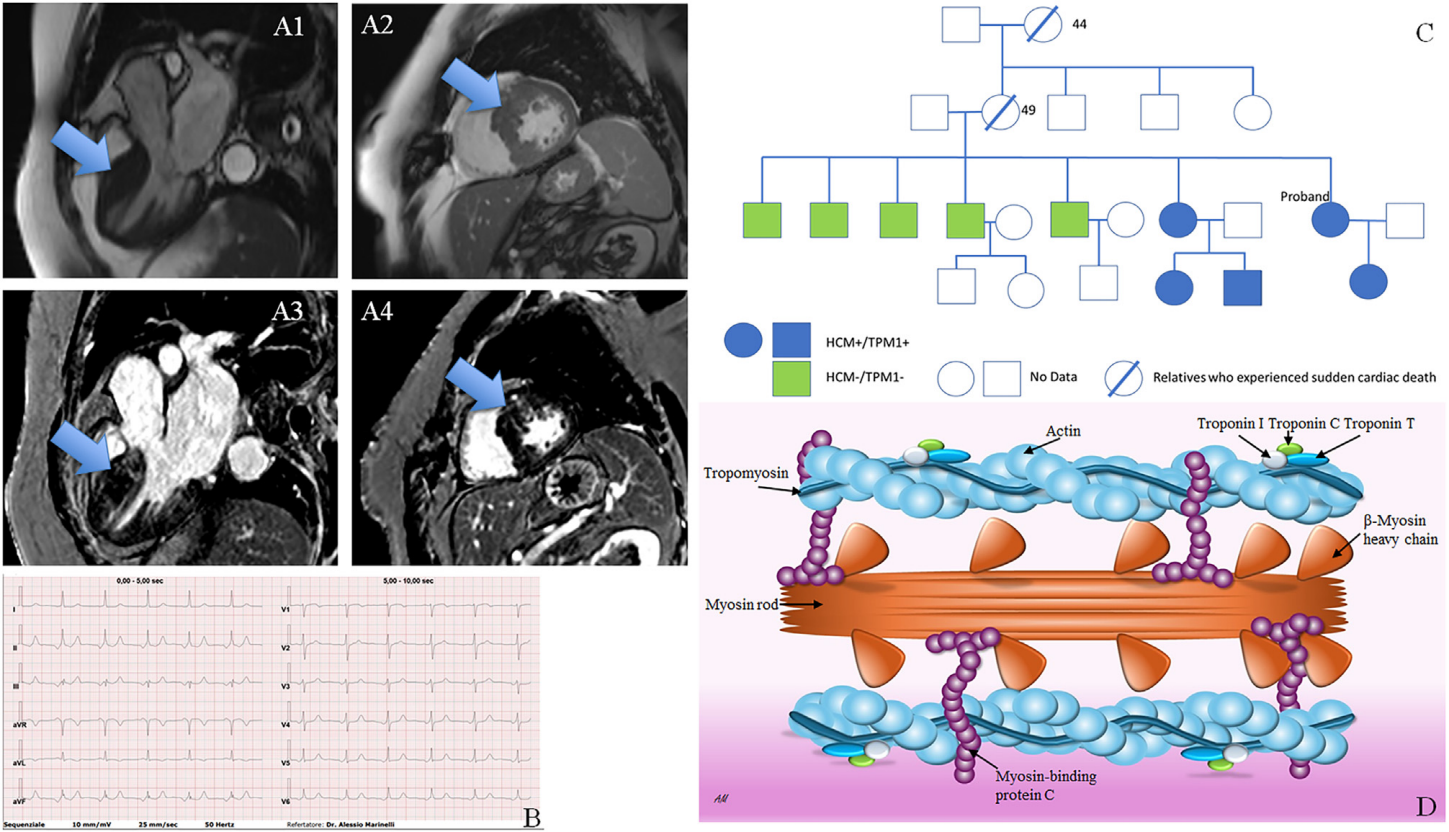
The TPM1 p. Tyr221Cys variant causing hypertrophic cardiomyopathy. **(A)** Steady-state free precession sequences show hypertrophic interventricular septum, especially at the basal and mid-level of the left ventricle (A1, A2); Late gadolinium enhancement characterized by a patchy distribution is identified in the hypertrophic segments (A3, A4). **(B)** Abnormal electrocardiogram. **(C)** Proband family tree. Blue elements are the patients affected by HCM and carriers of the TPM1 variant. The green elements are the brothers of the proband who do not carry the mutation and are disease-free. White elements are the people on whom no genetic data are available. The mother of the proband died suddenly at 49 years old, and the maternal grandmother at 44 years old. **(D)** Cardiac sarcomere with the major HCM disease-causing proteins/genes.

Customize exome panel was performed on the proband including all specific genes associated with cardiomyopathy, including genes responsible for cytoskeletal proteins, sarcomeric proteins, and nuclear envelope proteins. A total of 164 genes were investigated (see supplementary data). The extracted genomic DNA was processed using the Exome CG-CytoGenomics (Nonacus) capture assay kit and sequenced on Novaseq 6000 (Illumina) with an average coverage of 100X. The mapping and analysis were based on the UCSC hg19 reference sequence of the human genome. The variant TPM1 (NM_001018005.1):c662A > G (pTyr221Cys), up to now classified as a variant of uncertain significance, was identified in the proband and confirmed by Sanger sequencing.

The variant segregation study was extended to the other nine family members, with (*n* = 4) and without (*n* = 5) diagnosis of HCM. Family study segregation was performed by Sanger sequencing methodologies, using a specific primer set to target the region of interest c.662A > G after genomic DNA extraction. *In vitro* amplification of the exon 7 of TMP1 gene was performed by polymerase chain reaction using oligonucleotide primers (Fw 5′ CCATTTGATATCAGAGGTTCCATTA 3′ and Rv 3′ CGACCTTTGAATATTCCTGACTTGG 5′). Sequencing was performed using a BigDye Terminator Cycle Sequencing Kit (Applied Biosystems Inc., Foster City, CA, USA). The sequences were analyzed on an Applied Biosystems ABI 3500 capillary sequencer (https://www.thermofisher.com) in accordance with the manufacturer's manual.

We integrated three *in silico* analysis tools (PolyPhen-2; MutationTaster; PROVEAN) to predict the potential impact of the amino acid substitution predicting software. All of them concluded as “probably/possibly damaging” and “deleterious”. Further details can be seen in the supplemental material.

All four patients affected by HCM were found to also carry the TPM1 variant. The other family members without disease did not show the mutation.

Each analysis obtained informed consent from participants and approval from our institutional ethical review board (Ethics Committee of Verona and Rovigo, approval number 15871 of 13th March 2023).

The human *TPM1* gene is located on chromosome 15q22.2, and it codes 10 exons. This gene encodes tropomyosin 1 (TPM1), a thin filament protein expressed in both cardiac and fast skeletal muscle fibers. TPM1 is a member of a family of actin-binding proteins. Tropomyosin is the crucial determinant of the Ca^2+^-dependent regulation of actin-myosin interactions, mediated by the troponin complex calcium response because the position it assumes on the thin filament either blocks or exposes myosin-binding sites^3^ (Fig. 1D). Mutations in the *TPM1* gene have been identified in individuals with familial HCM, with a percentage of about 5% of all genotype-positive HCM patients.^2^

The missense variant c.662A > G described in this family, results in substituting a highly conserved tyrosine with cysteine in residue 221 of the protein (p.Tyr221Cys). This could be responsible for a local increase in positive charge in a relatively negatively charged region which could lead to structural distortions of a highly conserved region of the molecule, its binding to actin, and its position on the thin filament, causing alterations to the actin–myosin interactions and myofilament Ca^2+^ sensitivity. The final effect could be an accelerated rate of actin–myosin interactions, decreased relaxation, and increased force production.

Another very close mutation to the one we describe, the c.656A > T (p.D219V) on the TPM1 gene, has been recently very well described and analyzed by functional and *in vitro* studies on a case of a young girl with sudden cardiac death. The authors concluded that this variant significantly affected the properties of TPM1, causing a substantial increase in Ca^2+^ sensitivity and incomplete relaxation at low Ca^2+^ concentrations of the contractile apparatus of cardiac muscle, which is most likely a cause of the weakening of the TPM's interaction with actin in the “closed” state. Such changes are characteristic of genetic HCM, therefore this mutation is HCM-associated and likely pathogenic.^4^

To date, the variant c662A > G (p.Tyr221Cys) is classified as a variant of uncertain significance following the guidelines for the variants interpretation criteria of the American College of Medical Genetics and Genomics (ACMG Richards 2015). In the literature review, this variant was detected in at least one male patient affected by HCM^5^ (Nakashima et al, 2020). Moreover, this variant is absent from the public population database (gnomAD, http://gnomad.broadinstitute.org) and it is absent in the ClinVar database.

The genetic makeup of a patient, such as oligogenic studies or polygenic risk, can have a significant impact on the final manifestation and expression of a specific variant. Nevertheless, the proposed study begins with an extensive investigation of the proband by analyzing all the genes currently known to be associated with HCM in OMIM and ClinVar, focusing on all coding regions, as well as intronic regions and splicing regulatory sites within 10 bp of the intron/exon junction.

While our findings strongly suggest a “likely pathogenic” role based on genetic segregation, we acknowledge that experimental validation (such as functional assays, *in vitro* studies, or animal models) is essential to confirm the variant's functional impact.

## CRediT authorship contribution statement

**Alessio Marinelli:** Writing – review & editing, Conceptualization, Investigation, Writing – original draft, Methodology, Supervision, Visualization. **Giuliana Longo:** Conceptualization, Writing – original draft. **Vincenzo Cirigliano:** Data curation. **Esther Campopiano:** Investigation. **Clementina Dugo:** Conceptualization, Visualization. **Luca Ghiselli:** Methodology. **Andrea Chiampan:** Conceptualization, Visualization. **Gianluca Ferri:** Investigation. **Alessandro Costa:** Investigation. **Stefano Bonapace:** Investigation, Supervision. **Laura Lanzoni:** Investigation, Visualization. **Giulio Molon:** Supervision, Validation.

## Conflict of interests

The authors have no conflicts to disclose.
