# Supplementary material for: Clinical impact of the TPM1 p.Tyr221Cys variant causing hypertrophic cardiomyopathy
Source: Genes Dis. 2025 Feb 26;12(5):101575. doi: 10.1016/j.gendis.2025.101575 (PMC12166675; doi:10.1016/j.gendis.2025.101575)

**Material and method**

**NGS**: Customize exome panel was performed on the proband. The analysis was based on Whole Exome Sequencing (WES), more than 19.300 genes. A virtual Customized Exome Panel has been created including all specific genes associated to cardiomyopathy, including genes responsible for cytoskeletal, sarcomere, and nuclear envelope proteins. After a subscribe test requisition form during pretest genetic counselling to explain the limits and advantages of the test, the proband’s blood draw was collected.

The complete panel include all genes related to different cardiomyopathies and based on HPO gene AARS2, ABCC9, ACAD9, ACADVL, ACTA1, ACTC1, ACTN2, AGK, AGL, ALMS1, ALPK3, ANK2, ARSB, ATP5F1D, ATPAF2, BAG3, BRAF, CACNA1C, CBL, CDH2, COA5, COA6, COX10, COX14, COX15, COX2, COX6B1, COX7B, CPT2, CRYAB, CSRP3, DES, DMD, DNAJC19, DOLK, DSC2, DSG2, DSP, EMD, EPG5, EYA4, FAH, FASTKD2, FHL1, FHOD3, FKRP, FKTN, FLNC, FOXRED1, FXN, GAA, GATA6, GLA, GLB1, GUSB, HADHA, HADHB, HCN4, HFE, HGSNAT, HRAS, IDH2, IDS, IDUA, JPH2, JUP, KRAS, LAMP2, LDB3, LMNA, LRPPRC, LZTR1, MAP2K1, MAP2K2, MIB1, MLYCD, MMACHC, MRPL44, MUT, MYBPC3, MYH6, MYH7, MYL2, MYL3, MYPN, NAA5, NAGLU, NDUFA1, NDUFA10, NDUFA11, NDUFA2, NDUFA4, NDUFAF1, NDUFAF2, NDUFAF3, NDUFAF4, NDUFAF5, NDUFB11, NDUFB3, NDUFB8, NDUFS1, NDUFS2, NDUFS3, NDUFS4, NDUFS6, NDUFS7, NDUFS8, NDUFV1, NDUFV2, NEXN, NF1, NKX25, NONO, NRAS, NUBPL, PCCA, PCCB, PDLIM3, PET100, PKP2(98.02%), PLN, PNPLA2, PPA2, PPCS, PPP1CB, PPP1R13L, PRKAG2, PTPN11, RAF1, RASA2(99.75%), RBM2, RIT1, RYR2, SCN5A, SCO1, SCO2, SDHA, SDHAF, SDHD, SGCD, SGSH, SHOC2, SLC22A5, SLC25A20, SLC25A4, SOS1, SOS2, SURF1, TAZ, TCAP, TGFB3, TMEM126B, TMEM43, TMEM7, TNNC1, TNNI3, TNNI3K, TNNT2, TPM1, TSFM, TTN, TTR, UQCC2, VCL.

The extracted genomic DNA was processed using the Exome CG-CytoGenomics (Nonacus) capture assay kit sequenced on NGS Novaseq paltform, Illumina with an average coverage of 100X.The sequencing data are processed using the bioinformatics pipeline developed for this specific use. The mapping and analysis was based on the UCSC hg19 reference sequence of the human genome.

**Sanger sequencing**. To validate the positive variant identified via exome sequencing, Sanger sequencing was performed to confirm the presence or absence of these variant in the proband. Moreover, the variants segregation study was extended to other affected family members. The scope of extensions study was to define the clinical significant of the variant identified in the proband. Family study segregation was performed by Sanger sequencing methodologies, using a specific primer to amplify the region of interest c.662A>G after genomic DNA extraction.

After the NGS methodology, to confirm the presence of the variant in the proband we approached by Sanger Sequencing. We used the same protocol also for the other family members to perform the segregation study. In vitro amplification of the exon 7 of TMP1 gene was performed by polymerase chain reaction using oligonucleotide primers (Fw 5’CCATTTGATATCAGAGGTTCCATTA 3’ and Rv 3’CGACCTTTGAATATTCCTGACTTGG 5’). Sequencing was performed by using a BigDye Terminator Cycle Sequencing Kit (Applied Biosystems Inc., Foster City, CA, USA). The sequences were analyzed on an pplied Biosystems ABI 3500 capillary sequencer (https://www.thermofisher.com) in accordance with the manufacturer’s manual.

We integrated three in silico analysis tools to predict the potential impact of the amino acid substitution predicting software: prediction of functional effects of human SNPs (PolyPhen-2 v2.2.3r408 (http://genetics.bwh.harvard.edu/pph2/), MutationTaster (https://mutationtaster.org/ ) and Protein Variant Effect Analyzer (PROVEAN®) (http://provean.jcvi.org/index.php). PolyPhen-2 predicted the variant to be "probably damaging" with a HumVar score of 0.947 (sensitivity: 0.65; specificity: 0.92) and assessed it as "possibly damaging" in the context of the HumDiv score, which was 0.847 (sensitivity: 0.83; specificity: 0.93). Additionally, MutationTaster predicted the variant to be "disease-causing." The variant affects a highly conserved amino acid site across species, further supporting its potential pathogenicity. PROVEAN prediction score is -7.361, predicted to be “Deleterious”.


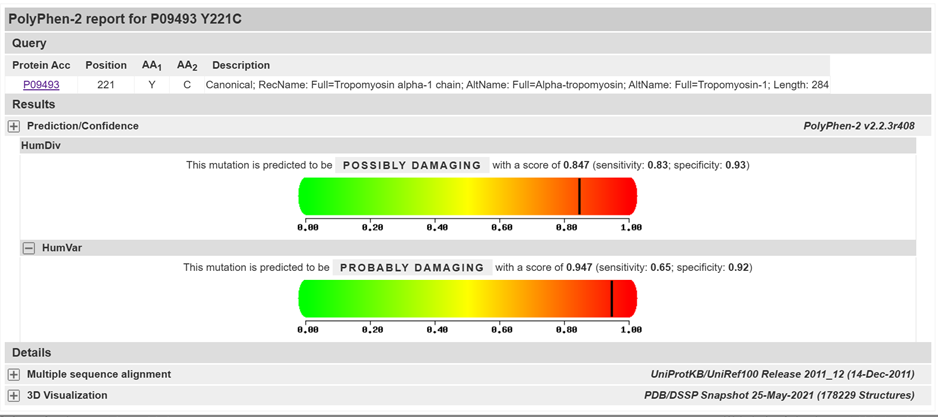

Supplement: Multimedia component 1 [file mmc1.docx]
